# Clinical Relevance of Prostate-Specific Membrane Antigen–Lipid Rafts Association and Membrane Protein Interactions in Prostatic Adenocarcinoma

**DOI:** 10.14740/wjon2833

**Published:** 2026-09-04

**Authors:** Awatef Ben Jemaa, Abdullah Hoter, Oumaima Khelifi, Sataa Sallami, Yassine Nouira, Ridha Oueslati, Hassan Y. Naim

**Affiliations:** a Unit IMEC-Immunology Microbiology Environmental and Carcinogenesis, Faculty of Science of Bizerte, University of Carthage, Bizerte, Tunisia; b Department of Biochemistry, University of Veterinary Medicine Hannover, Hannover, Germany; c Department of Urology, La Rabta Hospital, University Tunis Al Manar, Tunis, Tunisia

**Keywords:** PSMA, Prostate cancer, Lipid rafts, DPPIV, BiP

## Abstract

**Background:**

Lipid rafts or detergent-resistant membranes (DRMs) have played a fundamental role during prostate-specific membrane antigen (PSMA) activation by serving as platforms for internalization and cell signaling. In the current study, we aimed to evaluate the clinical significance of PSMA–lipid rafts association in prostate adenocarcinoma.

**Methods:**

The study was carried out on two normal prostate (NP), 11 benign prostate hyperplastic (BPH), and 10 prostate cancer (PC) samples. Immunohistochemical analysis for PSMA, CD34, and Ki67 was performed. Serum levels of prostate-specific antigen (PSA) were assayed by Immulite autoanalyzer. DRMs were isolated from LNCaP cells, NP, BPH, and PC tissues. Using Triton X-100, PSMA, dipeptidyl peptidase IV (DPPIV), binding immunoglobulin protein (BiP), and Flotillin-2 were analyzed in both soluble and insoluble fractions by Western blot analysis.

**Results:**

The findings demonstrate that PSMA is overexpressed in PC compared to normal and benign prostate tissues. The strongest staining of PSMA was membranous, with apical accentuation in neoplastic cells. Furthermore, upregulation of PSMA was concomitant with high expression of CD34 and Ki67 in malignant prostate. Our data indicate that PSMA, DPPIV, and BiP are distributed in the insoluble and soluble fractions of the analyzed PC samples. Nevertheless, the level of these membrane proteins is higher in the soluble fraction as compared to the DRMs in prostate adenocarcinoma.

**Conclusion:**

Overall, this preliminary study provides initial insights into the potential clinical relevance of the association between PSMA and lipid rafts in human prostatic adenocarcinoma. The observed co-distribution of PSMA with DPPIV and BiP in the DRM fractions of PC samples suggests a possible association among these membrane proteins that may contribute to PC progression. Although these findings are preliminary, they support the hypothesis that crosstalk between these proteins could occur. Further studies are required to elucidate the underlying molecular mechanisms and to determine whether targeting PSMA and its associated membrane partners may represent a promising therapeutic strategy.

## Introduction

Prostate cancer (PC) is a major global health concern, particularly for men over 50 years of age, and ranks as the second leading cause of cancer-related deaths among men worldwide [1]. PC exhibits high heterogeneity that presents a serious challenge for clinical PC management. Heterogeneity within prostate tumors, including variations in prostate-specific membrane antigen (PSMA) expression, can contribute to diverse phenotypes of PC cells [2, 3]. PC cells may be activated into either aggressive or less aggressive phenotypes, leading to patient resistance or sensitivity to therapies [2]. PSMA is a transmembrane protein that is significantly overexpressed in PC and in the neovasculature of many other cancer types [4]. Moreover, PSMA expression is correlated with more aggressive disease and worse outcomes and appears to enhance progression to castration-resistant PC [3, 5]. PSMA clusters upon activation at the cell surface of tumor and endothelial cells and undergoes internalization, making it a suitable target for passive immunotherapy [4, 5].

In LNCaP cells, antibody cross-linking of PSMA induces its activation and internalization. Activation of PSMA leads to subsequent MAPK activation (p38 and ERK1/2) concomitant with upregulation of the pro-inflammatory cytokine interleukin-6 (IL-6). These signaling pathways are linked to PC progression and resistance to hormonal therapy [6]. Like PSMA, dipeptidyl peptidase IV (DPPIV) is also an integral type II membrane glycoprotein that appears to accelerate PC progression, particularly to hormone-refractory disease [7]. Nevertheless, some studies report that DPPIV downregulation is linked to more advanced prostate tumor stage and the presence of metastasis [8].

In addition to PSMA, binding immunoglobulin protein (BiP), also known as glucose-regulated protein 78 (GRP78), which is normally located in the endoplasmic reticulum (ER), also appears on the surface of PC cells. Beyond its role in ER stress, BiP expression may be involved in signal transduction and may affect PC cell proliferation. Furthermore, higher BiP expression is linked to an increased risk of PC aggressiveness and recurrence [9, 10]. In previous studies, we demonstrated that lipid rafts play a key role in both the activation and internalization of PSMA [11, 12]. Indeed, PSMA distribution in the plasma membrane of LNCaP cells is not random and can be associated with lipid raft microdomains, which act as organizing centers for signaling, protein trafficking, and endocytosis [11, 12]. To get insight into the clinical significance of PSMA–lipid rafts association *in vivo* in prostatic adenocarcinoma, we investigated PSMA distribution into detergent-resistant membranes (DRMs) and its interaction with other membrane proteins such as DPPIV and chaperons like BiP in normal, benign, and malignant prostate samples.

## Materials and Methods

### Reagents and antibodies

Tissue culture material was purchased from Sarstedt, RPMI medium, Dulbecco’s Modified Eagle’s Medium and supplemented reagents (penicillin-streptomycin, fetal calf serum) were from PAA Laboratories. Triton X-100, proteinase inhibitors, protein A-sepharose, and bovine serum albumin were from Sigma. Acrylamide, TEMED, SDS, Tris, sodium chloride, potassium chloride, sodium hydrogen phosphate, potassium di-hydrogen phosphate, sodium deoxycholate, and polyvinylidene fluoride (PVDF) membranes were purchased from Carl Roth. The ECL plus Western Blotting Detection System was from Thermo Scientific. Coomassie blue G-250 was from Serva. PSA DPC Immulite assays were purchased from Diagnostic Products Corporation (DPC) (Immulite 1000; DPC, Los Angeles, CA). The primary antibodies used for immunohistochemistry analysis were mouse anti-human PSMA (3E6) and mouse anti-human CD34 (QBend10) from Dako (Dako, Glostrup, Denmark). CD34 antibody was used to label vessels in the prostate tissues. CD34 immunostaining was performed to evaluate microvascular density and provide an assessment of angiogenic activity in the prostate tissue samples. Angiogenesis is an important feature of tumor progression, and CD34 is a widely used endothelial marker for the identification and quantification of microvessels in tumor tissues. Mouse anti-human Ki67 was purchased from CUSABIO (CUSABIO Technology LLC, Houston, USA). Ki67 immunostaining was performed to assess cellular proliferative activity. Ki67 is a well-established proliferation marker that reflects the growth fraction of tumor cells and is commonly used to evaluate the biological aggressiveness of PC. The primary antibodies used for Western blot analysis included the anti-PSMA monoclonal antibody D2B, which recognizes a luminal epitope of PSMA and was produced in the Colombatti laboratory [6]. As previously shown, mAb D2B recognizes human PSMA with the same specificity and somewhat greater affinity than the PSMA-specific mAb J591 [6]. The anti-flotillin-2 and anti-DPPIV antibodies were from Santa Cruz Biotechnology. The anti-BiP antibody was purchased from Stressgen (Victoria, Canada).

### Subjects and samples

Prostates tissues were obtained from: (a) transurethral resections from 11 men (aged from 57 to 80 years) diagnosed clinically and histopathologically with benign prostatic hyperplasia (BPH); (b) radical prostatectomy from 10 men (aged from 58 to 81 years) diagnosed with PC; and (c) histologically two normal prostates (NPs) obtained at autopsy (8–10 h after death) from men (aged 22 and 24 years) without histories or reproductive, endocrine, or related diseases. All pathological, clinical, and personal data were anonymized and separated from any personal identifiers. The study protocol was reviewed and approved by the appropriate institutional ethics committee. For BPH and PC samples, written informed consent was obtained from all participants prior to their inclusion in the study. All the procedures followed were examined and approved by the Hospital of La Rabta of Tunis and the Hospital of Charles Nicolle of Tunis (Tunisia) (06/222).

Immediately after collection, tissue specimens were transported under appropriate conditions and processed for histological evaluation and biochemical analysis. Following surgical excision, each tissue specimen was divided into two portions. One portion was fixed in 10% neutral buffered formalin for histopathological evaluation and immunohistochemistry, while the remaining portion was snap-frozen in liquid nitrogen and stored at −80 °C for subsequent biochemical and molecular analyses.

Peripheral blood samples were collected in EDTA tubes before any specific cancer treatment (patients) and transurethral resections or radical prostatectomy surgery. Peripheral blood (5 mL) was collected from each participant using standard sterilization procedures, and serum components were centrifuged and immediately stored in a freezer at −80 °C until further analysis of PSA levels.

### Immunohistochemistry

Immunochemical procedure specificity was checked using negative controls. For negative controls, tissues of each type (NP, BPH, and PC) were incubated with blocking peptides (Santa Cruz Biotechnology, CA, USA) at the same immunoglobulin concentration used for each antibody.

For immunohistochemical evaluation, tissue specimens were fixed in 10% formaldehyde, dehydrated, and embedded in paraffin. Paraffin blocks were sectioned at a thickness of 3 µm and processed using the NovoLink™ Polymer Detection System (Novocastra Laboratories Ltd., Newcastle, UK). Sections were deparaffinized, rehydrated through a graded ethanol series, and rinsed in deionized water. Antigen retrieval was performed by incubating the sections in 0.1 M citric acid buffer (pH 6.0) at 98 °C for 20 min in a water bath. Slides were then allowed to cool for an additional 20 min and subsequently washed with deionized water. Endogenous peroxidase activity was blocked by treating the sections with Peroxidase Block for 5 min. All incubation steps were conducted at room temperature, and each step was followed by two washes in Tris-buffered saline (TBS) for 5 min each. To minimize non-specific binding of the primary antibody, sections were treated with Protein Block for 5 min. Primary antibodies were then applied at dilutions of 1:50 for PSMA and 1:100 for CD34 and Ki67, prepared in antibody diluent (Dako, Glostrup, Denmark), and incubated at room temperature for 30 min. Subsequently, sections were exposed to Post Primary Block for 30 min to reduce non-specific binding of the polymer. This was followed by incubation with NovoLink™ Polymer for 30 min. Peroxidase activity was visualized by treating the sections with 3,3′-diaminobenzidine (DAB) working solution for 5 min. Finally, the slides were counterstained with hematoxylin and mounted.

Immunolabeling was quantitatively evaluated and compared across all tissue groups for each of the three antibodies. For each prostate specimen, six histological sections were randomly selected for analysis to minimize sampling bias. From each section, five microscopic fields were randomly chosen and imaged at × 40 magnification. Digital images were analyzed using an automated image analysis system (Motic Images Advanced, version 3.2; Motic China Group Co., China). For each field, the region of interest (ROI), corresponding to the relevant prostatic tissue, was manually delineated. Immunostaining was quantified by measuring the mean optical density (OD) per unit area within the ROI, which reflects the intensity of the specific immunohistochemical signal. For every immunopositive section, a corresponding negative control section from an adjacent serial section was analyzed, and the OD of the control was subtracted from that of the stained section. Mean OD values were calculated for each prostate sample, and data were expressed as mean ± standard error of the mean (SEM) for each prostatic group (NP, BPH, and PC). These mean values were then used for statistical comparisons among the different tissue groups and antibodies.

Analyses were independently performed by two observers, yielding consistent results. The number of sections analyzed was determined through iterative sampling to identify the minimum number required to achieve the lowest SEM.

### PSA immunoassays

Serum PSA concentrations were measured using the PSA DPC Immulite assay (Immulite 1000; DPC, Los Angeles, CA), following the manufacturer’s protocol. This method is a solid-phase, two-site sandwich immunoassay that employs both monoclonal and polyclonal anti-PSA antibodies. Signal detection was achieved through enhanced chemiluminescence. According to DPC reference standards, a PSA value of 4 ng/mL was considered within the normal range. The exact serum PSA values were unavailable for the autopsy cases. Therefore, the PSA value was reported as < 4 ng/mL for normal cases.

### Cell culture

The PSMA-positive LNCaP human PC cell line was from ATCC. Cells were maintained *in vitro* by serially passaging in RPMI-10% FBS at 37 °C in a humidified atmosphere of 5% CO_2_. LNCaP cell line plating was performed on poly-D-lysine coated tissue culture plasticware (10 µg/mL). Cells were grown to confluence in RPMI-Medium containing 2 g/L glucose in the presence of fetal calf serum (10% v/v) and penicillin-streptomycin (100 U/mL and 0.1 mg/mL, respectively).

### Preparation of DRMs

LNCaP cells of 70% confluence were washed twice in ice-cold phosphate-buffered saline (PBS) and solubilized in PBS containing 1% (w/v) detergent (Triton X-100) supplemented with a mixture of protease inhibitors (PIs). Cells were homogenized with a Luer-21 Gauge needle 20 times/mL and then maintained on ice for 2 h. In the case of prostate tissues, each sample was homogenized in the extraction buffer (2 mM Tris, 50 mM mannitol and PI mix) and were solubilized in PBS containing 1% (w/v) detergent (Triton X-100) supplemented with a mixture of PIs. Afterwards, samples were pre-centrifuged for 15 min at 10,000 × g and cell debris was discarded followed by a centrifugation at 100,000 × g, 4 °C for 90 min (Beckman Optima LE-80, SW 55Ti-rotor). The supernatant and pellet obtained, corresponding to the soluble and insoluble fractions respectively, were separately analyzed. The pellet fractions were lysed overnight in a lysis-buffer containing 25 mM Tris, 50 mM sodium chloride, 0.5% (w/v) Triton X-100, and 0.5% (w/v) sodium-deoxycholate supplemented with a mixture of PIs. On the next day, the protein amounts of all fractions were determined using Bradford protein assay (Bio-Rad) and 25 mg protein of each fraction was analyzed by SDS/PAGE and Western blotting.

### SDS-PAGE, Western blotting, and fluorography

Protein samples were resolved by SDS-PAGE on 8% polyacrylamide slab gels according to LaemmLi under reducing conditions using 10 mM dithiothreitol (DTT). The gels were blotted onto PVDF membranes (240 mA, 1.5 h). The membrane was blocked in 5% skimmed milk in PBS and 0.1% Tween 20 (PBST). The membranes were treated with either one of the following primary antibodies: anti-PSMA mAb D2B (1 µg/µL), anti-DPPIV mAb (1 µg/µL), anti-BiP mAb (1 µg/µL), and anti-flotillin-2 mAb (0.2 µg/µL) for 1 h at room temperature. The blots were washed three times with PBST and subjected to further treatment with the secondary antibody, anti-mouse IgG conjugated to horseradish peroxidase (0.4 µg/ µL; Thermo Fisher Scientific). All the antibodies were used at a dilution of 1:5,000. The protein bands were visualized via enhanced chemiluminescent peroxidase substrate and documented with a ChemiDoc MP™ (Bio-Rad, Munich, Germany). Digital images obtained were quantified using ImageLab™ software. Digital images of the immunoblots were analyzed by densitometric analysis using Image Lab™ software (Bio-Rad Laboratories, Hercules, CA, USA). Western blot bands were quantified by densitometric analysis using image analysis software. The values from independent experiments were expressed as mean ± standard deviation (SD). Statistical comparisons among the NP, BPH, PC, and LNCaP groups were performed using one-way analysis of variance (ANOVA) or Student’s *t*-test if only two groups were compared, with a P value < 0.05 considered statistically significant.

### Statistical analysis

All statistical analyses were carried out using GraphPad Prism software (version 5.0; GraphPad Software). Data normality was evaluated with the Shapiro–Wilk test. Categorical variables were summarized as frequencies or percentages and analyzed using Fisher’s exact test. Continuous data were reported as mean with range or as median with interquartile range (IQR), depending on their distribution. Group comparisons for normally distributed continuous variables were performed using the unpaired Student’s *t*-test, whereas the Mann–Whitney U test was applied to non-normally distributed variables. When comparisons involved more than two groups, one-way ANOVA was used. For all analyses, a P value less than 0.05 was considered statistically significant.

## Results

A total of 11 patients with BPH and 10 patients with PC disease admitted in the urology department were enrolled in the study. In addition, two NP obtained at autopsy served as controls. The clinic and biological data of these patients are summarized in Table 1. The median age of BPH patients was 73 (57–80) years, while the median age of PC patients was 74 (58–81) years (Table 1). Regarding the grade of the PC patients, there was no patient with grade < 6, four (40%) were classified as Gleason Score (GS) 6–7, and six (60%) were with grade 8–10 (P = 0.007). Moreover, the PC group had a significantly higher median (IQR) sera PSA level (89.4 (53.5–935) ng/mL vs. 10.33 (6.23–22.21) ng/mL; P < 0.0001) as compared to BPH group (Table 1).

**Table 1:** Comparison of Clinical and Biological Parameters in Patients With BPH and PC

Parameters	Normal prostate (n = 2)	BPH (n = 11)	PC (n = 10)	P-value
Age, median (range)	23 (22–24)	73 (57–80)	74 (58–81)	0.38
Gleason Score, n (%)				
< 6	-	-	0 (0%)	
6–7	­-	­-	4 (40%)	0.007*
8–10	-	-	6 (60%)	
PSA (ng/mL), median (IQR)	< 4	10.33 (6.23–22.21)	89.4 (53.5–935)	< 0.0001*
IHC analysis				
PSMA (% OD)				
n (%)	2 (100)	4 (36)	10 (100)	< 0.0001*
Median (IQR)	2.84 (0.86–4.83)	17.25 (16.27–19.27)	43.63 (4.23–51.65)	0.036*
CD34 (% OD)				
n (%)	2 (100)	11 (100)	10 (100)	1.0
Median (IQR)	2.59 (1.73–3.46)	8.81 (6.61–13.39)	18.38 (15.52–18.85)	0.0001*
Ki67 (% OD)				
n (%)	2 (100)	11 (100)	10 (100)	1.0
Median (IQR)	19.5 (18.37–20.63)	19.15 (18.82–21.45)	24.21 (19.65–26.65)	0.0143*

*P < 0.05. BPH: benign prostate hyperplasia; IHC: immunohistochemical; IQR: interquartile range; OD: optical density; PC: prostate cancer; PSA: prostate-specific antigen; PSMA: prostate-specific membrane antigen.

**Figure 1:**
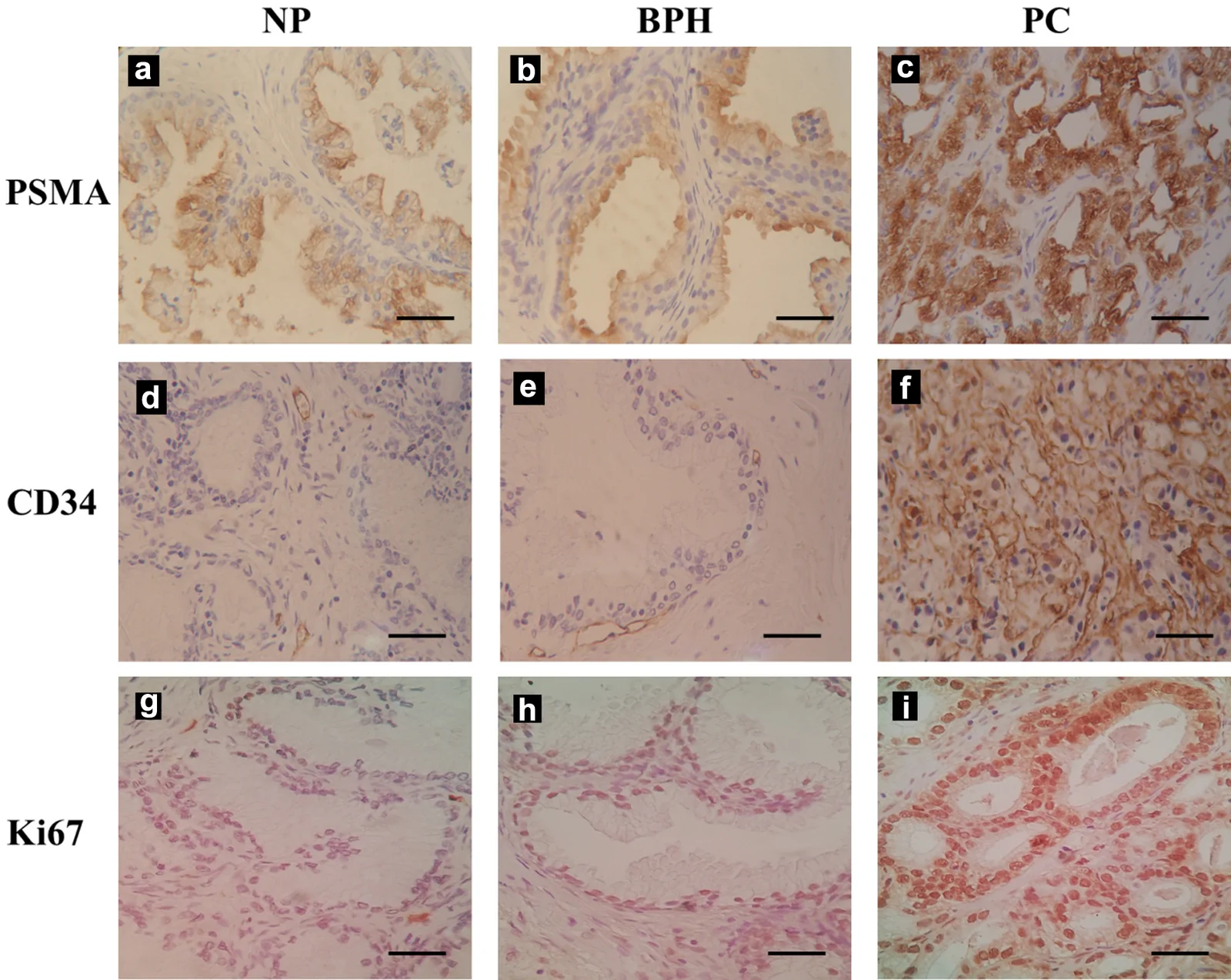
Prostate-specific membrane antigen (PSMA), CD34, and Ki67 immunohistochemical staining patterns in normal prostate (NP), benign prostate hyperplasia (BPH), and prostate cancer (PC). PSMA revealed low to moderate staining in luminal cells of NP (a) and BPH (b), while it exhibited strong cytoplasmic and membranous staining in neoplastic cells of PC samples (c). CD34 showed low to moderate immunoexpression in endothelial cells of NP (d) and BPH (e), whereas strong staining is found in endothelial cells of prostate adenocarcinoma samples (f). Ki67 showed low to moderate expression in the nuclei of cells in NP (g) and BPH (h), while strong staining is observed in nuclei of neoplastic cells in PC tissues (i). Scale bar: 100 µm.

To determine the expression of the prostate tumor-associated antigen, PSMA, an immunohistochemistry analysis was performed. Immunoreactivity to PSMA appeared in the two cases of NP, 36% of BPH, and 100% of PC samples (Table 1). In NP and BPH samples, PSMA was exclusively expressed in the cytoplasm and membrane of luminal epithelial cells. Likewise, immunoreaction to PSMA was restricted to the cytoplasm and membrane of tumor cells of PC specimens. Interestingly, the strongest staining pattern of PSMA was membranous with accentuation in apical of neoplastic cells of vast majority of PC (Fig. 1). Immunostaining intensity to PSMA was higher in PC (median (IQR) 43.63 (4.23–51.65)) than in NP and BPH (median (IQR) 2.84 (0.86–4.83) and 17.25 (16.27–19.27), respectively; P = 0.036)) (Table 1 and Fig. 1). In order to analyze the degree of vascularization in prostate specimens, we detected immunohistochemically the marker of endothelial cells, CD34. Immunoreactivity to CD34 was found in endothelial cells of all NP, BPH, and PC samples (Table 1 and Fig. 1). Similarly to PSMA, immunostaining intensity of CD34 was higher in PC (median (IQR) 18.38 (15.52–18.85)) as compared to NP and BPH samples (2.59 (1.73–3.46) and 8.81 (6.61–13.39), respectively; P = 0.0001) (Table 1 and Fig. 1). To further assess the proliferation activity, we analyzed the proliferation index Ki67 in normal, benign, and malignant prostate samples. As denoted in Table 1, Ki67 staining appeared in all cases of NP, BPH, and PC. Furthermore, Ki67 immunoexpression was restricted to the nuclei of cells in normal, benign, and malignant prostate tissues (Fig. 1). The OD revealed higher immunoexpression of Ki67 in prostatic adenocarcinoma (median (IQR) 24.21 (19.65–26.65)) as compared to NP and BPH samples (median (IQR) 19.5 (18.37–20.63) and 19.15 (18.82–21.45), respectively, P = 0.0143) (Table 1 and Fig. 1).

Next, we analyzed the expression of PSMA and different lipid raft and non-raft markers within human prostate tissue and LNCaP cells using Western blot. Digital images of the immunoblots were analyzed by densitometric analysis using Image Lab™ software (Bio-Rad Laboratories, Hercules, CA, USA). Western blot bands were quantified by densitometric analysis using image analysis software. The values from independent experiments were expressed as mean ±SD. Statistical comparisons among the NP, BPH, PC, and LNCaP groups were performed using one-way ANOVA or Student’s *t*-test if only two groups were compared, with a P value < 0.05 considered statistically significant.

**Figure 2:**
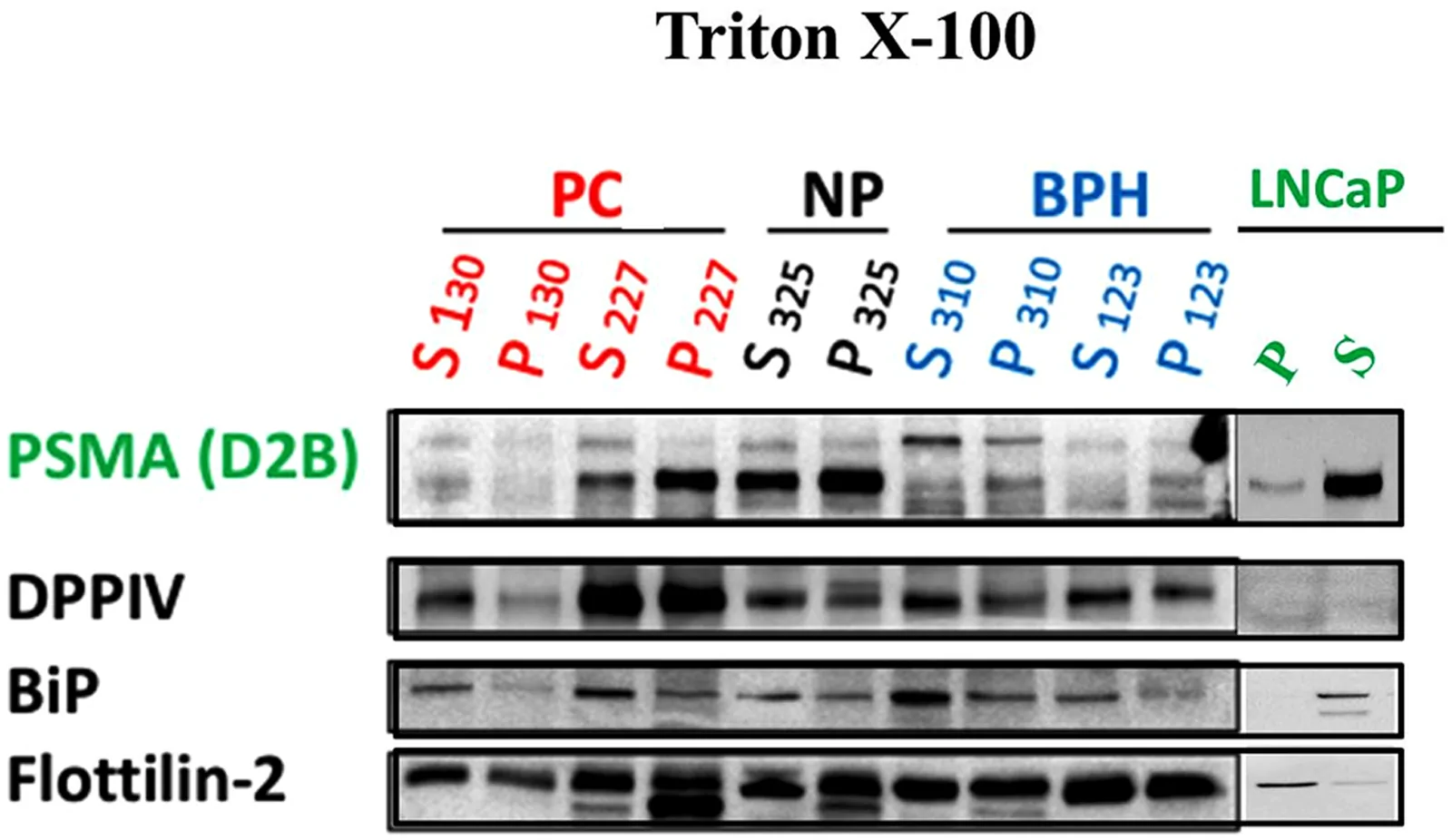
Western blot analysis of prostate-specific membrane antigen (PSMA), dipeptidyl peptidase IV (DPPIV), binding immunoglobulin protein (BiP), and Flottinlin-2 after prostate samples and LNCaP cells were solubilized with 1% Triton X-100 (w/v) in phosphate-buffered saline (PBS). Lysates were subjected to centrifugation at 100,000 × g for 90 min in order to separate the insoluble (P) detergent-resistant membrane (DRM)-fraction and the soluble (S) material. After centrifugation the insoluble fraction (P) was solubilized with a lysis-buffer containing 0.5% (w/v) Triton X-100 and 0.5% (w/v) sodium-deoxycholate. Equal amounts of total protein loaded for each lane on 8% polyacrylamide gel electrophoresis. The lanes showing a band correspond to a positively stained prostate of each group. NP: normal prostate; BPH: benign prostate hyperplasia; PC: prostate carcinoma; P: pellet (insoluble fraction); S: soluble fraction.

**Figure 3:**
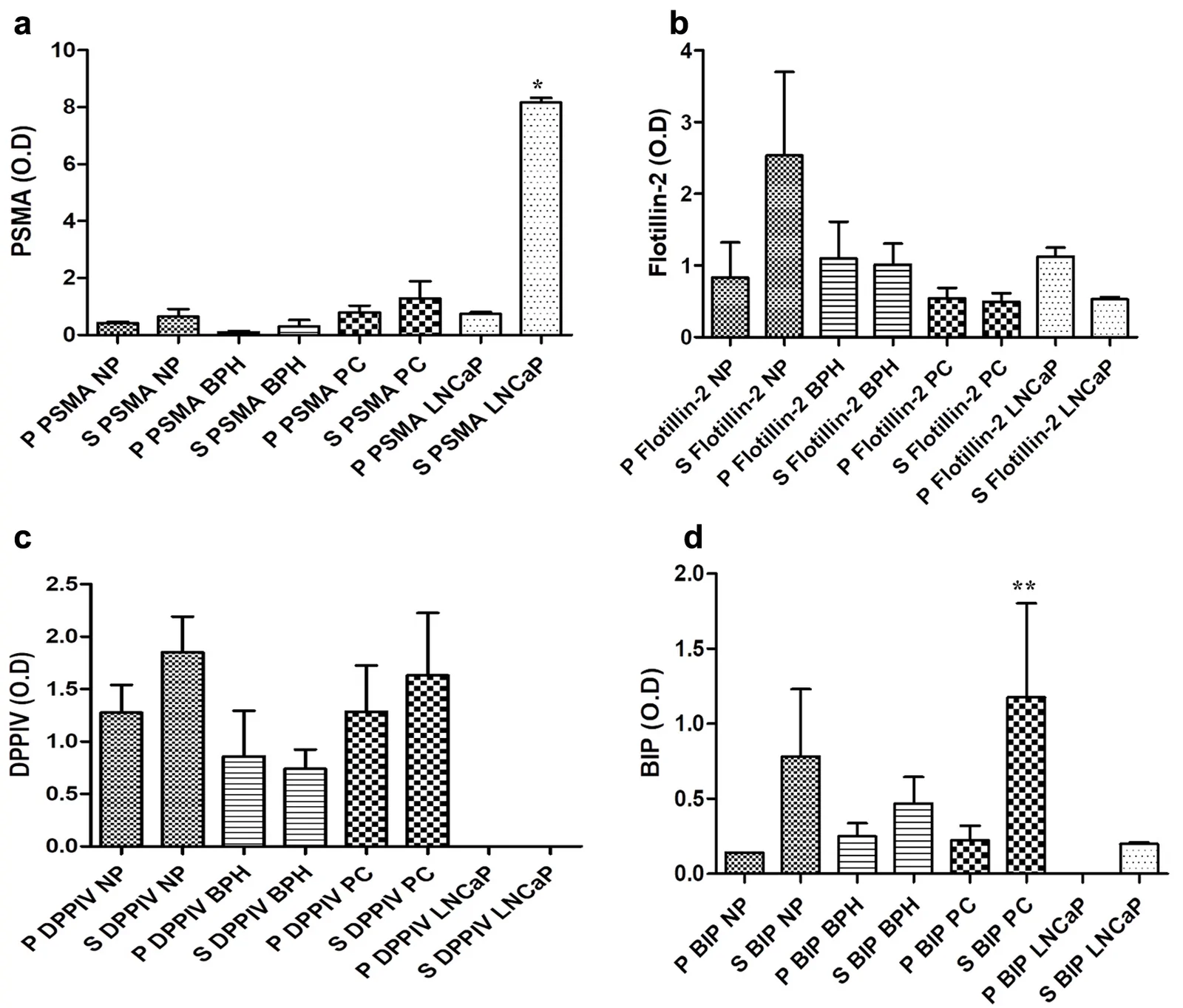
Comparison of immunostaining intensities (measured as average optical density ± standard deviation (SD)) in Western blot analysis in normal prostate (NP), benign prostate hyperplasia (BPH), prostate carcinoma (PC), and LNCaP cells for prostate-specific membrane antigen (PSMA) (a), Flottilin-2 (b), dipeptidyl peptidase IV (DPPIV) (c), and binding immunoglobulin protein (BiP) (d) after prostate samples and LNCaP cells were solubilized with 1% Triton X-100 (w/v) in phosphate-buffered saline (PBS). Average optical densities were evaluated only in patients showing positive immunoreactions. P: pellet; S: supernatant. Significance was accepted when P < 0.05. *P < 0.05, **P < 0.01.

To get insight into the interaction of PSMA with proteins in lipid rafts *in vivo* in human prostate tissue, DRMs were extracted from normal, benign, and malignant prostate samples after solubilization with Triton X-100. The supernatant and pellet obtained, corresponding to the soluble and insoluble fractions respectively, were separately analyzed. Thus, we further analyzed the distribution of PSMA, different lipid raft and non-raft markers within the soluble and non-soluble fractions. As shown in Figure 2, PSMA is predominantly soluble in Triton X-100 for LNCaP cells, while is partially insoluble in this DRMs for normal, benign, and malignant prostate tissues. Although there were no significant differences for PSMA levels between the insoluble and soluble fractions, we found a slight increase in PSMA expression in the supernatant compared to the pellet fraction in NP, BPH, and PC samples (Fig. 3a). One of the approaches to identify LRs in cellular preparations is to assess the expression levels of Flotillin-2, a widely used raft marker protein. Figure 2 demonstrates that Flotillin-2 was also partially found in the soluble fractions of Triton X-100-DRMs for LNCaP cells, NP, BPH, and PC samples. As shown in Figure 3b, Flotillin-2 is more abundant in the soluble fraction than the pellet in NP tissues. However, no differences were found for Flottilin-2 between the two fractions in BPH and PC samples. For LNCaP cells, the expression level of Flotillin-2 is higher in the insoluble than the supernatant fraction (Fig. 3b). Similar to PSMA, DPPIV protein is also partially insoluble in Triton X-100-DRMs for normal, benign, and prostatic adenocarcinoma tissues (Fig. 2). As illustrated in Figure 3c, we observed a slight increase in the DPPIV expression in the soluble compared to the non-soluble fraction for normal and neoplastic prostate. For BPH samples, we found a minor increase of DPPIV expression in the pellet compared to the supernatant. Nevertheless, there were no significant differences of DPPIV expression between the two fractions in each prostate type. Regarding LNCaP cells, DPPIV is not expressed either in the supernatant and pellet of these PC cells (Figs. 2 and 3c). We further investigated the interaction between the ER chaperone BiP and Triton X-100-DRMs in prostate samples and LNCaP cell line. As shown in Figure 2, BiP is partially insoluble in Triton X-100 for prostate tissues and LNCaP cells. However, BiP expression is higher in the soluble than in insoluble fraction for NP, BPH, PC samples and LNCaP cells. Interestingly, the expression levels of BiP are increased about threefold in the supernatant as compared to the pellet in prostatic adenocarcinoma tissues (Fig. 3d). Importantly, unlike LNCaP cell line, Western blot analysis revealed that prostate samples exhibit heterogeneous expression of PSMA, lipid raft and non-raft markers in each prostate group (NP, BPH, and PC). Overall the intensity of expression of these protein markers varies between prostate samples within each of the three prostate types.

## Discussion

Although the role of lipid rafts or DRMs during the activation and internalization of PSMA has been well studied [11, 12], the clinical significance of PSMA–lipid raft association in prostate adenocarcinoma remains elusive. In the present work, we show that PSMA is highly expressed in PC samples compared with normal and benign prostate tissues. Furthermore, our results revealed that PSMA overexpression in malignant prostate cases is concomitant with high expression of the endothelial cell marker CD34. These findings support the role of PSMA as a protumor protein that may enhance tumor aggressiveness and angiogenesis in PC [4, 5]. Interestingly, our data show that PSMA overexpression in PC is associated with high expression of the proliferation marker Ki67. Ki67 is a valuable prognostic marker that helps predict progression and recurrence in patients with PC [13]. Consistent with our findings, previous studies have shown that higher Ki67 expression correlates with more aggressive PC features, such as advanced T stage, lymph node metastasis, and poorer survival outcomes [13, 14]. Accordingly, our results provide further evidence supporting the involvement of PSMA in PC progression, promoting tumor cell proliferation and aggressiveness [6]. Moreover, PSMA staining revealed both cytoplasmic and membranous localization in luminal cells of the prostatic gland. However, the strongest staining pattern was observed at the apical membrane of neoplastic cells in the majority of PC cases. This apical localization is particularly important for PSMA activation and internalization. Notably, the microtubule cytoskeleton plays a key role in PSMA endocytosis and recycling within the endosomal compartment [12, 15].

To confirm the immunohistochemistry results of PSMA immunoexpression, Western blot analysis was performed in prostate samples and LNCaP cells. Western blot results revealed PSMA expression in normal, benign, and malignant prostate tissues, with PC and BPH samples exhibiting the highest levels compared with normal prostate tissue. PSMA was initially identified in LNCaP cells using an immunoprecipitation technique [16]. Our study also confirmed PSMA expression in LNCaP cells. In addition to PSMA, we analyzed another transmembrane glycoprotein, DPPIV, in prostate samples and LNCaP cells. DPPIV is expressed in immune cells (especially T lymphocytes) and in several tumors, including prostate adenocarcinoma [17]. Similar to PSMA, DPPIV is expressed in epithelial cells of the prostate gland and is highly upregulated in PC, particularly in advanced and hormone-refractory stages [7, 17]. Our results showed DPPIV expression in most BPH and PC samples, with no significant difference between the two groups. In contrast to our findings, previous studies on human prostate samples reported elevated DPPIV expression in PC compared with noncancerous tissues [7, 18]. Unlike PSMA, DPPIV expression was not detected in LNCaP cells in our study. Conversely, a previous study by Wen et al reported higher DPPIV protein expression in LNCaP cells compared with PC3 and DU145 cells [19].

Experiments using LNCaP cells showed that, following internalization, PSMA translocates to the endosomal compartment [12, 15]. BiP (GRP78) is a crucial ER chaperone protein involved in PC aggressiveness and recurrence [20]. As a marker of the ER [21], we assessed BiP expression in normal, benign, and malignant prostate tissues. We detected BiP immunoexpression in LNCaP cells and most prostate samples, with the highest expression observed in PC and BPH cases. This chaperone protein, which is normally localized in the ER, can also be expressed on the surface of PC cells, where it correlates with poor prognosis and therapeutic resistance [9, 10]. Previous studies have demonstrated that PSMA is partially localized in lipid rafts of LNCaP cells, where it forms signaling complexes that contribute to prostate tumor progression [11, 12]. One approach to identify lipid rafts in LNCaP cells is to assess the scaffold protein flotillin-2 [22]. Our data show that flotillin-2 is expressed in the majority of normal, benign, and PC tissues. Moreover, consistent with previous observations, we found that LNCaP cells also exhibit flotillin-2 expression [11, 12].

The role of lipid rafts in PSMA trafficking and function has been well documented in LNCaP cells [11, 12], whereas little is known about the clinical significance and potential impact of PSMA–LR interaction in prostate adenocarcinoma. To gain insight into PSMA–LR association *in vivo*, normal, benign, and malignant prostate tissues were lysed and solubilized using Triton X-100. Supernatant and pellet fractions, corresponding to the soluble and insoluble fractions, respectively, were collected and analyzed. During its transport, PSMA interacts with different membrane microdomains, including lipid rafts, also known as DRMs [11, 12]. The presence of PSMA in lipid rafts is particularly relevant in prostate tumor cells, where it may contribute to cell surface signaling and be involved in maintaining cellular survival and proliferation [11, 12]. Therefore, understanding PSMA’s localization within lipid rafts is important for grasping its role in signaling, trafficking, and endocytosis in PC cells. Our results clearly demonstrated expression of PSMA in both soluble and insoluble fractions of each NP, BPH, and PC samples. Interestingly, there was slight increase in the intensity of PSMA levels in the soluble fraction compared to the detergent-insoluble fraction in prostatic adenocarcinoma tissues. Our data show that PSMA is partially enriched in the detergent-insoluble, lipid-rich raft fraction, meaning that a subpopulation of PSMA molecules is raft-associated.

To identify lipid rafts in prostate tissue preparations, we assessed the expression levels of flotillin-2, a widely used raft marker protein [22]. Flotillin-2 is also partially present in the soluble fractions of Triton X-100–resistant DRMs in LNCaP cells, as well as in NP, BPH, and PC samples. The PSMA–lipid raft association is crucial for PSMA activation and its subsequent internalization in PC cells. The interaction of PSMA with lipid rafts and cytoskeletal proteins plays a key role in its internalization process, suggesting that the cellular environment influences PSMA function in prostate tumor progression and invasiveness [11, 12]. As lipid rafts concentrate growth factor receptors, Src-family kinases, and adaptor proteins, the partitioning of PSMA into these rafts enables its interaction with these molecules and modulates signaling pathways, including IL-6/STAT3, NF-κB, and MAPKs (p38 and ERK1/2). These pathways are associated with PC progression and resistance to hormonal therapy [6, 11].

Moreover, PSMA localization to lipid rafts may enhance PC cell signaling, facilitate internalization, and influence how PC cells respond to therapies [11, 12]. Our results also indicate that a large portion of PSMA resides in the detergent-soluble fraction in PC samples. This non–raft-associated PSMA pool appears to remain active as a peptidase but may be less involved in signaling [11, 12]. In LNCaP cells, the highest PSMA expression is found in the soluble fraction. This finding is consistent with prior studies reporting that mature PSMA is completely soluble in Triton X-100 and partially insoluble in the detergent Lubrol WX [11, 12]. The solubility of PSMA varies according to the detergent used for lipid raft isolation and cell membrane lysis. In LNCaP cells, mature, complex-glycosylated PSMA distributes as Lubrol WX–insoluble dimers that are enriched in lipid raft fractions [11, 12]. Homodimerization of PSMA is a prerequisite for its association with Lubrol WX–resistant DRMs. PSMA associates with Lubrol WX–resistant DRMs in the Golgi compartment concurrent with acquisition of its native, complex-glycosylated form. PSMA first enters the lipid rafts as a precursor glycoform in the Golgi. After processing and dimerization, mature dimeric PSMA homodimers reach the plasma membrane within these Lubrol WX–insoluble lipid/protein complexes, where the majority remains at steady state [11, 12]. Triton X-100, however, completely solubilizes mature PSMA, indicating that its association with lipid rafts depends on the detergent properties as well as the protein’s post-translational modifications and quaternary structure, which are established during Golgi processing and dimerization [11, 12].

DPPIV is a type II transmembrane glycoprotein expressed in several tumors, including PC [7]. We investigated the interaction of PSMA and DPPIV with lipid rafts in normal, benign, and malignant prostate samples. Western blot analysis showed that DPPIV is partially insoluble in Triton X-100–resistant DRMs in NP, BPH, and prostatic adenocarcinoma tissues. Similar to PSMA, there was a slight increase in DPPIV expression in the soluble compared with the insoluble fraction in neoplastic prostate samples. Notably, unlike PSMA, DPPIV expression was low in LNCaP cells and was not detected in either insoluble or soluble cellular fractions by Western blot. Malignant prostate tissue generally contains high levels of DPPIV, which is associated with more advanced tumor stage, higher Gleason scores, presence of metastasis, and larger tumor size [7]. However, DPPIV may also act as a tumor suppressor and can be downregulated during progression to castration-resistant PC [8]. These findings suggest a dual role of DPPIV during PC progression. DPPIV has multiple interaction partners, including the raft marker caveolin-1 [23]. In microvascular endothelial cells, the association of PSMA with caveolin-1 plays a key role in its internalization [24]. In line with these observations, our results support the notion that PSMA and DPPIV can interact with lipid rafts, which in turn may influence PC progression.

BiP expression is often elevated in PC cells, correlating with poor prognosis and therapeutic resistance by supporting cell survival under ER stress [9, 10]. Similar to PSMA, BiP isolated from DRM fractions was found in both pellet and supernatant fractions of prostate samples. Importantly, BiP is more abundant in the supernatant than in the insoluble fraction of prostatic adenocarcinoma samples. However, Triton X-100 completely solubilizes BiP in LNCaP cells. These data indicate that PSMA and BiP are distributed similarly between insoluble and soluble fractions in PC samples. These findings suggest a potential association between these proteins within lipid raft–enriched membrane domains but do not establish direct co-localization molecular interaction.

Consistent with PSMA, higher BiP expression is linked to increased risk of PC recurrence and poorer overall survival [9, 10]. Prior studies have reported that PC cells exhibit BiP expression on their surface, a characteristic also observed for PSMA [25, 26]. Consequently, co-expression of PSMA and BiP in both detergent-insoluble and soluble fractions of PC samples may suggest potential interaction or crosstalk between the PC biomarker and this chaperone protein. BiP is known to be expressed on the surface of prostate tumor cells, where it can participate in signal transduction with other membrane proteins [25, 27]. In line with these findings, our data suggest that the presence of BiP in lipid rafts is particularly relevant in PC cells, where it might contribute to cell surface signaling involving the membrane protein PSMA.

One of the most relevant results of our study is that PSMA, DPPIV, and BiP isolated from DRM fractions are heterogeneously expressed across PC samples. PC heterogeneity is considered a result of the emergence of different phenotypes of PC cells in response to transduction of different signals [2, 28]. In light of these previous observations, our data suggest that PSMA–lipid rafts association is dynamic and involves different other membrane proteins within these rafts like DPPIV and BiP that could modulate PSMA expression through various signal transduction in cellular context-specific manner.

A limitation of the present study is the relatively small sample size, particularly in the NP group. Although the observed differences provide preliminary evidence of altered protein expression, larger, independent cohorts are required to validate these findings and determine their clinical significance. Since CD31 is the most specific and currently preferred marker for vascularization, future studies incorporating CD34-based microvessel density assessment and additional endothelial marker CD31 would provide further characterization of tumor vascularization.

### Conclusions

Altogether, our findings provide preliminary evidence of an association between PSMA and lipid rafts in human prostatic adenocarcinoma, offering initial insights into their potential clinical relevance. Furthermore, the observed co-distribution of PSMA with DPPIV and BIP in DRM fractions of PC samples suggests a possible crosstalk among these membrane-associated proteins in PC cells. However, these observations should be considered exploratory and require confirmation in larger patient cohorts and additional functional studies to establish their biological and clinical significance. As the relationship within lipid rafts between membrane proteins PSMA, DPPIV, and BiP appears to be complex, understanding the molecular mechanisms underlying their regulation will require careful investigation.

## Data Availability

The data supporting the findings of this study are available from the corresponding author upon reasonable request.
